# Heme Oxygenase-1 Regulates Zearalenone-Induced Oxidative Stress and Apoptosis in Sheep Follicular Granulosa Cells

**DOI:** 10.3390/ijms25052578

**Published:** 2024-02-23

**Authors:** Yina Li, Yujin Gao, Dan Yao, Zongshuai Li, Jiamian Wang, Xijun Zhang, Xingxu Zhao, Yong Zhang

**Affiliations:** 1 College of Veterinary Medicine, Gansu Agriculture University, Lanzhou 730070, China; lyn9097@163.com (Y.L.); gyj1234561202@163.com (Y.G.); 15776502994@163.com (D.Y.); 17899319313@163.com (J.W.); zhangxijun0325@163.com (X.Z.); 2 Gansu Key Laboratory of Animal Generational Physiology and Reproductive Regulation, Lanzhou 730070, China; lizsh2010@163.com; 3 State Key Laboratory of Grassland Agro-Ecosystems, Key Laboratory of Grassland Livestock Industry Innovation, Ministry of Agriculture and Rural Affairs, Grassland Agriculture Engineering Center, Ministry of Education, College of Pastoral Agriculture Science and Technology, Lanzhou University, Lanzhou 730020, China

**Keywords:** zearalenone, follicular granulosa cell, oxidative stress, apoptosis, HMOX1

## Abstract

Zearalenone (ZEA) is a common non-steroidal estrogenic mycotoxin found in a range of animal feeds and poses a serious threat to the reproductive health of farm animals and humans. However, the mechanism underlying ZEA-induced reproductive toxicity in sheep remains unknown. Granulosa cells are crucial for egg maturation and the fertility of female sheep. In this study, we aimed to examine the impact of different ZEA concentrations on sheep follicular granulosa cells and to elucidate the potential molecular mechanism underlying ZEA-induced toxicity using transcriptome sequencing and molecular biological approaches. Treating primary sheep follicular granulosa cells with different concentrations of ZEA promoted the overproduction of reactive oxygen species (ROS), increased lipid peroxidation products, led to cellular oxidative stress, decreased antioxidant enzyme activities, and induced cell apoptosis. Using transcriptome approaches, 1395 differentially expressed genes were obtained from sheep follicular granulosa cells cultured in vitro after ZEA treatment. Among them, heme oxygenase-1 (HMOX1) was involved in 11 biological processes. The protein interaction network indicated interactions between HMOX1 and oxidative and apoptotic proteins. In addition, N-acetylcysteine pretreatment effectively reduced the ZEA-induced increase in the expression of HMOX1 and Caspase3 by eliminating ROS. Hence, we suggest that HMOX1 is a key differential gene involved in the regulation of ZEA-induced oxidative stress and apoptosis in follicular granulosa cells. These findings provide novel insights into the prevention and control of mycotoxins in livestock.

## 1. Introduction

Zearalenone (ZEA), an endocrine disruptor with estrogen-like effects, is a mycotoxin predominantly produced by *Fusarium* spp. strains that are widely present in cereals and agricultural products; it is one of the most prevalent mycotoxins contaminating feedstuffs [1,2]. ZEA is chemically stable and resistant to high temperatures; therefore, it is not inactivated and can be stabilized during feed processing [1,3]. When animals consume feed contaminated with ZEA, this toxin exhibits various toxic effects. ZEA and its metabolites reportedly cause various hazards to humans and animals, including reproductive toxicity, cytotoxicity, genotoxicity, and immunotoxicity, and their reproductive effects have received widespread attention [4,5]. Estrogenic effects of ZEA can lead to various reproductive disorders in livestock and may even lead to high estrogen levels, thereby hindering reproduction in livestock and likely causing hyperestrogenic syndrome in humans [2,6]. Experimental evidence suggests that ZEA impedes egg and embryo development and interferes with spermatogenesis in animals, thereby affecting fertility [7,8,9]. ZEA exposure increases phosphatidylcholine or phosphatidylethanolamine levels and depletes hemolysin phosphatidylcholine, thereby impeding follicular development and oocyte maturation [10]. It also induces autophagy, apoptosis, and oxidative stress by affecting the structure and functions of the organelles, specifically the mitochondria, endoplasmic reticulum, and lysosomes, and disrupts embryonic development, thereby affecting oocyte maturity and follicle formation [11,12,13].

Follicular granulosa cells are the largest cell population in the follicles and play an important role in oocyte development and maturation [14]. Granulosa cells are also involved in physiological processes, such as the maintenance of oocyte meiosis and follicular atresia [15,16]. Recent studies have demonstrated that different doses of ZEA significantly inhibit the growth of chicken granulosa cells, leading to apoptosis by activating the mitochondrial apoptotic pathway [17]. Another previous report indicates that ZEA causes follicular granulosa cell apoptosis, endoplasmic reticulum stress, and oxidative stress and affects steroid hormone production in pigs [18]. Because ZEA is widespread in cereals and produces toxic effects in a wide range of animals, with sensitivities varying among species [19], reproductive disorders due to its effects are frequently reported in farm animal species [6]. However, few studies have been reported on the mechanism of reproductive toxicity of ZEA in sheep.

Sheep comprise a major species in the livestock industry. Their feed primarily includes maize silage, maize stover, and alfalfa, which are susceptible to mycotoxin contamination if not stored properly [20]. Early studies have shown that ZEA affects ovulation rates and lambing percentages in female sheep [21,22,23]. Accidental ingestion of ZEA-contaminated feed by sheep may result in reproductive disorders or ZEA accumulation throughout the food chain, thereby affecting human health. ZEA impairs the morphology of primordial and primary follicles in sheep ovaries and is involved in oocyte autophagy; however, the exact mechanism underlying the toxicity remains unknown [24].

This study aimed to explore the effects of ZEA in different concentrations on sheep follicular granulosa cells and to provide experimental evidence for the potential molecular mechanism underlying ZEA-induced toxicity in granulosa cells in sheep. The results are expected to complement the experimental data gaps in the study of ZEA poisoning mechanisms in animals.

## 2. Results

### 2.1. ZEA Decreases the Viability of Sheep Follicular Granulosa Cells

**Figure 1:**
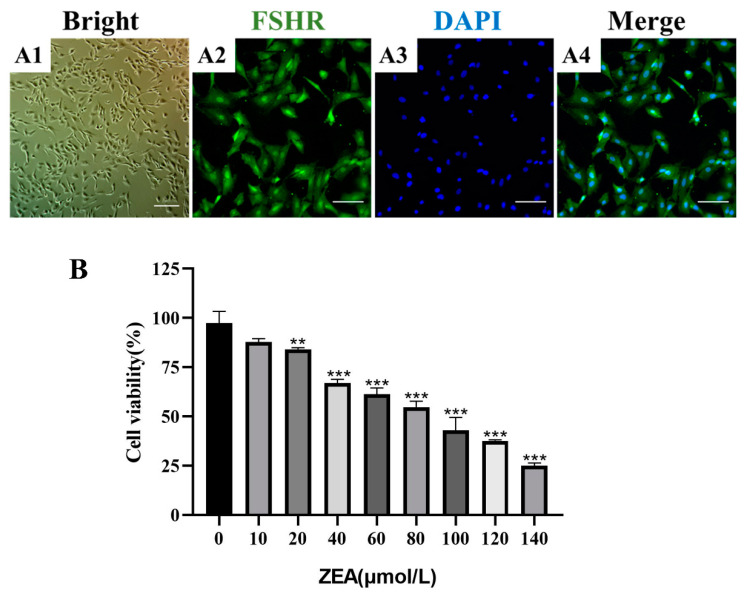
Identification of primary sheep follicular granulosa cells and cell viability analysis with ZEA treatment. (**A1**–**A4**) Identification of primary sheep follicular granulosa cells using immunofluorescence to detect FSHR. In order, brightfield (**A1**), FSHR (**A2**), DAPI (**A3**), and merger (**A4**). Bar = 100 µm. (**B**) Cell viability analysis of sheep follicular granulosa cells treated with ZEA (10–140 μM) (*n* = 5). Data are presented as means ± SD. ** represents *p* < 0.01, *** represents *p* < 0.001 compared with the control group.

The primary follicular granulosa cells isolated from sheep ovaries were identified using immunofluorescence for follicle-stimulating hormone receptor (FSHR), a marker for follicular granulosa cells (Figure 1A), and almost all investigated cells exhibited positive staining. To investigate the toxic effects of ZEA on sheep follicular granulosa cells, CCK-8 reagent was used to detect cellular activity. As shown in Figure 1B, compared with the control group, the ZEA-treated group exhibited a dose-dependent reduction in follicular granulosa cell activity, and the cell viability was significantly reduced with 20 μM ZEA (*p* < 0.001). Moreover, approximately 50% cell survival was noted after treatment with 80 μM ZEA. Therefore, we selected 20, 40, and 80 μM of ZEA for subsequent experiments.

### 2.2. ZEA Induces Oxidative Stress in Sheep Follicular Granulosa Cells

**Figure 2:**
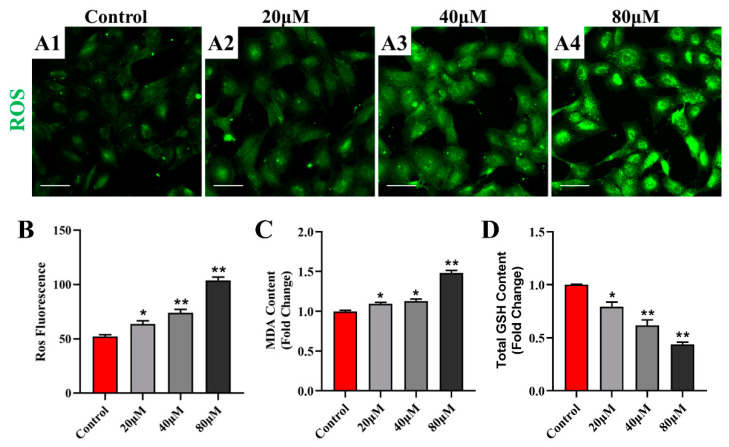
Detection of oxidative indicators in sheep follicular granulosa cells with ZEA treatment. (**A1**–**A4**) ROS level in follicular granulosa cells after ZEA treatment for 24 h. In order, control group (**A1**), 20 μM ZEA group (**A2**), 40 μM ZEA group (**A3**), and 80 μM ZEA group (**A4**). Bar = 100 µm. (**B**) Histogram of ROS fluorescence intensity (*n* = 3). (**C**) MDA content of follicular granulosa cells after ZEA treatment for 24 h (*n* = 3). (**D**) Total GSH content of follicular granulosa cells after ZEA treatment for 24 h (*n* = 3). Data are presented as means ± SD. * represents *p* < 0.05, ** represents *p* < 0.01 compared with the control group.

Excessive reactive oxygen species (ROS) induce oxidative stress. The fluorescence intensity in the follicular granulosa cells in Figure 2A indicated that different concentrations of ZEA resulted in a significant dose-dependent increase in ROS levels (Figure 2B). To further investigate the effects of ZEA on oxidative stress, malondialdehyde (MDA) and total glutathione (GSH) levels were simultaneously measured. Compared with that in the control group, the MDA content was significantly higher in the ZEA group (Figure 2C); meanwhile, the total GSH content was significantly lower in the ZEA group (Figure 2D). In summary, ZEA caused oxidative stress in sheep follicular granulosa cells.

### 2.3. ZEA Induces Apoptosis in Sheep Follicular Granulosa Cells

**Figure 3:**
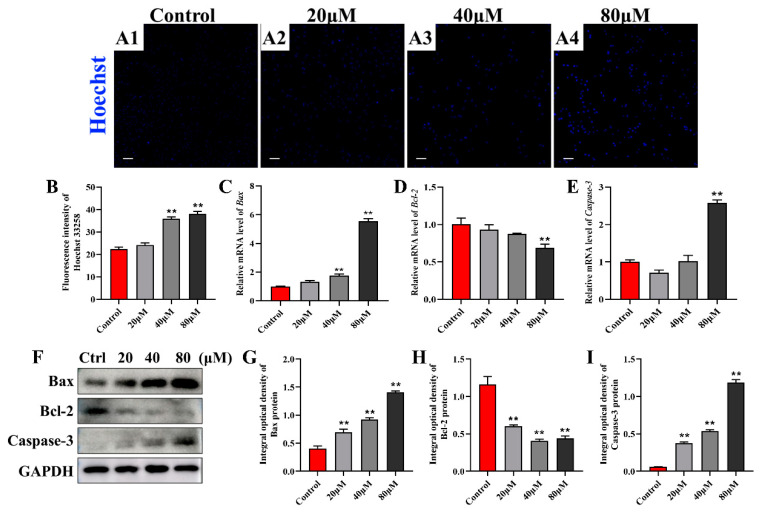
Detection of apoptosis in sheep follicular granulosa cells after ZEA treatment. (**A1**–**A4**) Hoechst 33258 staining of sheep follicular granulosa cells after ZEA treatment for 24 h. In order, control group (**A1**), 20 μM ZEA group (**A2**), 40 μM ZEA group (**A3**), and 80 μM ZEA group (**A4**). Bar = 100 µm. (**B**) Histogram of Hoechst 33258 fluorescence intensity (*n* = 3). (**C**–**E**) The expression levels of Bax (**C**), Bcl2 (**D**), and Caspase3 (**E**) mRNA in sheep follicular granulosa cells were detected using qRT-PCR (*n* = 3). (**F**–**I**) The expression levels of Bax (**G**), Bcl2 (**H**), and Caspase3 (**I**) protein in sheep follicular granulosa cells were detected using Western blot (*n* = 3). The expression of GAPDH was used as an endogenous control. Data are presented as means ± SD. ** represents *p* < 0.01 compared with the control group.

Excessive ROS accumulation induces oxidative damage leading to apoptosis. To investigate whether ZEA-induced oxidative damage leads to apoptosis in sheep follicular granulosa cells, we used Hoechst 33528 staining. The results of Hoechst 33528 staining analysis for detecting apoptosis revealed a significantly increased number of apoptotic cells in the ZEA groups at different concentrations (Figure 3A), and the fluorescence intensity was substantially higher in the 40 and 80 μM ZEA groups (Figure 3B). In addition, we examined the expression levels of apoptosis-related genes and proteins using quantitative real-time polymerase chain reaction (qRT-PCR) and Western blot. Compared with those in the control group, the mRNA and protein expression levels of Bax and Caspase3 gradually increased in the ZEA groups at different concentrations, while the expression level of Bcl2 gradually decreased. Among them, the difference between the 80 μM ZEA group and the control group was significant (Figure 3C–I). Taken together, the results suggest that ZEA induces apoptosis in sheep follicular granulosa cells.

### 2.4. Initial Validation of Transcriptome Data

**Figure 4:**
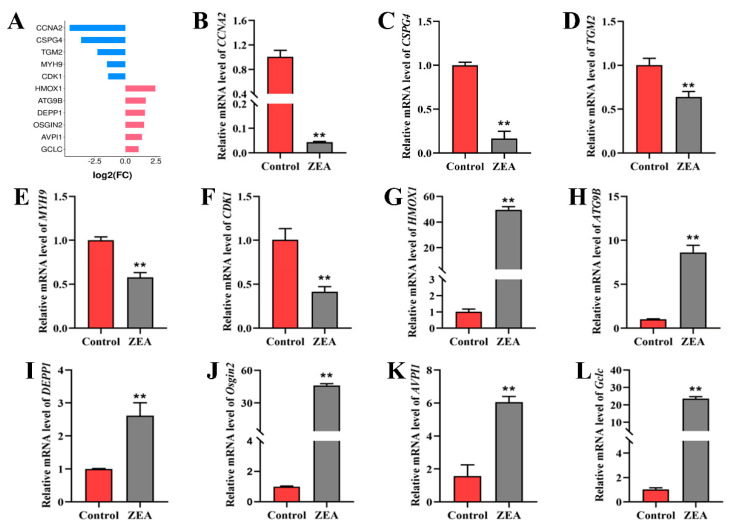
Initial validation of transcriptome data. (**A**) Histogram of log2 values for CCNA2, CSPG4, TGM2, MYH9, CDK1, HMOX1, ATG9B, DEPP1, OSGIN2, AVPI1, and GCLC in transcriptome data. (**B**–**L**) The expression levels of CCNA2 (**B**), CSPG4 (**C**), TGM2 (**D**), MYH9 (**E**), CDK1 (**F**), HMOX1 (**G**), ATG9B (**H**), DEPP1 (**I**), OSGIN2 (**J**), AVPI1 (**K**), and GCLC (**L**) mRNA in sheep follicular granulosa cells treated with 80 μM ZEA for 24 h were detected using qRT-PCR (*n* = 3). The expression of GAPDH was used as an endogenous control. Data are presented as means ± SD. ** represents *p* < 0.01 compared with the control group.

**Figure 5:**
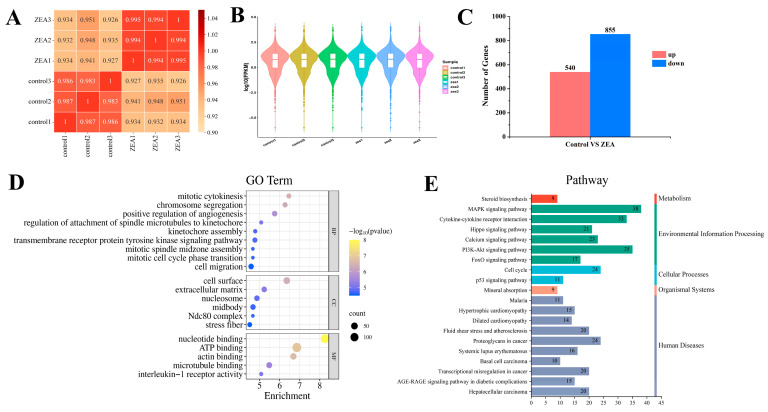
Visualization of transcriptome data. (**A**) Correlation heatmap of transcriptome data. Each group comprised three biological replicates. (**B**) Violin diagrams of six transcriptome samples. (**C**) Histogram of the number of differentially expressed genes in the transcriptome. (**D**) Top 20 of GO terms enrichment analysis of DEGs. (**E**) Top 20 of KEGG enrichment analysis of DEGs. DEGs, differentially expressed genes.

*CCNA2*, *CSPG4*, *TGM2*, *MYH9*, *CDK1*, *HMOX1*, *ATG9B*, *DEPP1*, *OSGIN2*, *AVPI1*, and *GCLC* were randomly selected for qRT-PCR. Compared with the control group, the mRNA expression levels of *CCNA2*, *CSPG4*, *TGM2*, *MYH9*, and *CDK1* in the ZEA group decreased (Figure 4B–F), whereas the levels of *HMOX1*, *ATG9B*, *DEPP1*, *OSGIN2*, *AVPI1*, and *GCLC* increased (Figure 4G–L). The results of qRT-PCR were consistent with those in the transcriptome (Figure 4A), indicating the validity and reliability of the transcriptome data. According to the correlation heatmap (Figure 5A), the correlation coefficient between the samples was ≥0.94. The violin plot shows that the samples were not degraded or abnormal (Figure 5B), which further established the transcriptome data were valid and reliable.

### 2.5. Enrichment Analysis of GO Terms and KEGG Pathway

The numbers of differentially expressed upregulated (log2FC ≥ 1, q < 0.05) and downregulated (log2FC ≤ −1, q < 0.05) genes in each comparison group were counted, and 540 upregulated and 855 downregulated genes were screened (Figure 5C). GO analysis of differentially expressed genes (DEGs) was performed (Figure 5D). The GO entries in the top 20 enrichment rankings primarily belonged to biological processes, which were related to cell–cell interactions and the cell cycle. KEGG analysis of DEGs (Figure 5E) primarily affected pathways related to environmental information processing such as the PI3K-Akt and MAPK signaling pathways and cytokine–cytokine receptor interaction.

### 2.6. Screening of HMOX1 as an Important DEG Based on Transcriptome Data

**Figure 6:**
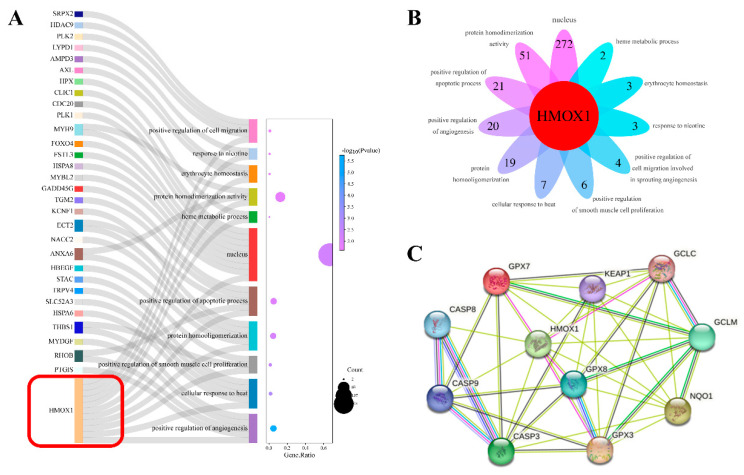
Important DEGs identified based on transcriptome data. (**A**) Sankey diagram of DEGs’ enrichment analysis. (**B**) Venn diagram of the 11 GO terms selected from transcriptomics. (**C**) Protein–protein interaction (PPI) networks with HMOX1 as the core protein. DEGs, differentially expressed genes; GO, gene ontology.

In total, 1395 DEGs were identified using transcriptomics (Figure 5C). The results demonstrated that HMOX1 was involved in 11 biological processes (Figure 6B), suggesting that it plays a crucial role in these biological processes. A Sankey diagram was created to further demonstrate the enrichment of DEGs in these 11 biological processes (Figure 6A). Subsequently, the protein–protein interaction (PPI) networks were constructed using STRING with HMOX1 as the core protein; the PPI network (Figure 6C) comprised 11 nodes and 79 edges. These included three apoptotic proteins, caspase3, caspase8, and caspase9, and included seven oxidation-related proteins, Gpx3, Gpx7, Gpx8, Keap1, Nqo1, Gclc, and Gclm, which showed that HMOX1 interacted with apoptosis- and oxidation-related proteins. It had been hypothesized that HMOX1 is an important candidate gene for oxidative stress and apoptosis induced by ZEA in sheep follicular granulosa cells.

### 2.7. HMOX1 Involves in ZEA-Induced Oxidative Stress and Apoptosis in Follicular Granulosa Cells

**Figure 7:**
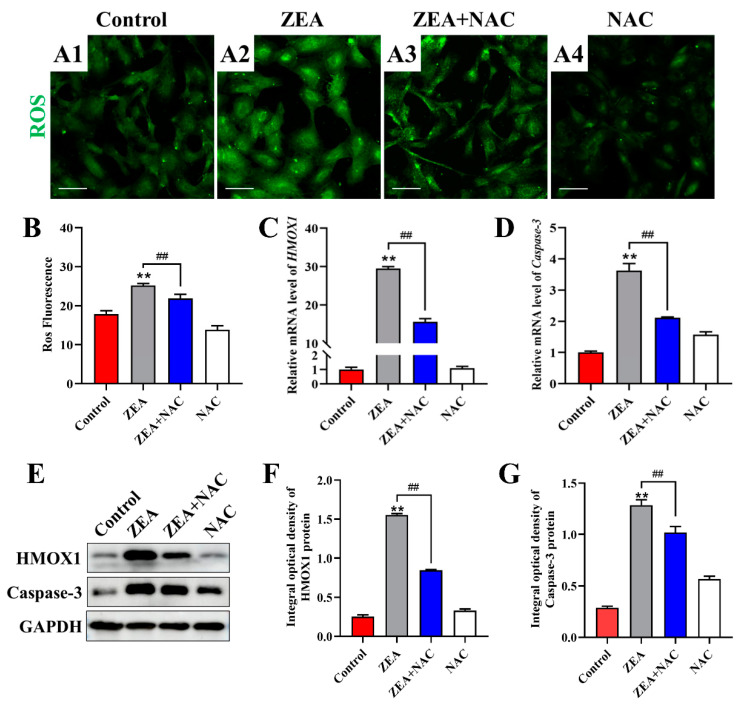
Exploring the role of HMOX1 in ZEA-induced oxidative stress and apoptosis. (**A1**–**A4**) ROS level in follicular granulosa cells pretreated with NAC for 6 h and then treated with 80 μM ZEA for 24 h. In order, control group (**A1**), ZEA group (**A2**), ZEA and NAC group (**A3**), and NAC group (**A4**). Bar = 100 µm. (**B**) Histogram of ROS fluorescence intensity (*n* = 3). (**C**,**D**) The expression levels of HMOX1 (**C**) and Caspase3 (**D**) mRNA in sheep follicular granulosa cells were detected using qRT-PCR (*n* = 3). (**E**–**G**) The expression levels of HMOX1 (**F**) and Caspase3 (**G**) protein in sheep follicular granulosa cells were detected using Western blot (*n* = 3). The expression of GAPDH was used as an endogenous control. Data are presented as means ± SD. ** represents *p* < 0.01 compared with the control group. ## represents *p* < 0.01 compared with the ZEA group.

N-acetylcysteine (NAC) pretreatment exhibits antioxidant effects based on the elimination of excess ROS. We pretreated follicular granulosa cells with 4 mM NAC for 6 h to enhance the cellular antioxidant capacity, followed by ZEA treatment for 24 h. We observed that the NAC-pretreated group had significantly reduced ROS levels compared with the ZEA-treated group (Figure 7A,B). To further elucidate the role of HMOX1 in ZEA-induced oxidative damage and apoptosis in follicular granulosa cells, the mRNA and protein expression levels of HMOX1 and Caspase3 were examined. Compared with the ZEA-treated group, elimination of excess ROS through NAC treatment decreased the mRNA and protein expression levels of HMOX1 as well as the expression of apoptosis-associated protein caspase3 (Figure 7), suggesting that HMOX1, as a key differential gene, might influence ZEA-induced oxidative stress and apoptosis.

**Figure 8:**
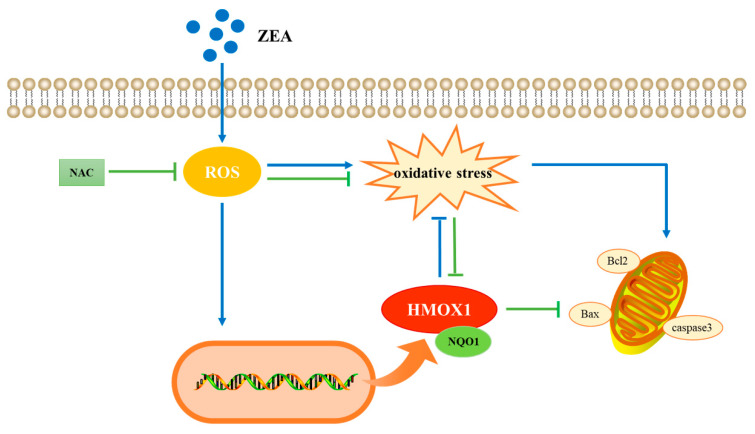
Conceptual diagram of the mechanisms underlying the role of HMOX1 in the regulation of ZEA-induced oxidative stress and apoptosis.

Finally, we inferred a conceptual diagram of HMOX1 regulates zearalenone-induced oxidative stress and apoptosis in sheep follicular granulosa cells (Figure 8).

## 3. Discussion

ZEA is one of the most common mycotoxins. Numerous studies have shown that it affects the reproductive capacity of animals [25,26,27]. The widespread distribution of ZEA in animal feed exposes farm animals to its toxic effects, which is a global public health problem [19]. Accumulation of ZEA in the food chain can be hazardous to human health and may even affect the pathogenesis and prevalence of estrogen-related carcinogenicity [28,29]. Both in vitro and in vivo tests confirmed that ZEA causes reproductive system damage in mice, pigs, cattle, and chickens [17,30,31,32]; however, few studies have focused on the mechanism underlying the toxicity of ZEA in sheep. In this study, we researched the toxic effects of ZEA on follicular granulosa cells of sheep cultured in vitro and the potential mechanism of action, providing theoretical support for ZEA-mediated reproductive damage.

Granulosa cells are important constituent units of the ovary and play an essential role in the maturation of animal oocytes and the production of steroid hormones [33]. CCK-8 results show that ZEA dose-dependently inhibited the activity of sheep follicular granulosa cells. The cell survival rate was approximately 50% at a ZEA concentration of 80 μM, which was therefore selected for subsequent experiments. Most intracellular ROS production under normal conditions is related to mitochondria [34]. Previous studies have shown that ZEA affects the activity, number, structure, and function of mitochondria and also affects the electron transport chain [35,36,37,38]. ZEA-induced mitochondrial damage may be an important pathway for promoting ROS production [39]. Excessive ROS production causes cellular oxidative stress, leading to an increase in lipid peroxidation products and a decrease in antioxidant indices [40]. Our results demonstrated a significant increase in intracellular ROS and MDA levels with increasing ZEA concentration; however, the levels of the antioxidant enzyme GSH significantly decreased. This indicates that ZEA promoted ROS production in sheep follicular granulosa cells and, to some extent, disrupted the balance of the intracellular antioxidant system, resulting in cellular oxidative damage. This result is consistent with the finding that ZEA promoted ROS generation, which induced oxidative stress and autophagy in dairy goat support cells [41]. This further confirmed that an increase in ROS is an important factor affecting cellular physiological processes.

Apoptosis plays an important role in normal cellular physiological processes [42]. ROS mediates lipid peroxidation, which interacts with membrane receptors and transcription factors to stimulate the activation of intrinsic and extrinsic apoptotic signaling pathways, which induce apoptosis through interactions with the Bcl2 family of mitochondrial proteins [43]. In this study, ZEA downregulated Bcl2 expression and upregulated Bax and Caspase3 expression at mRNA and protein levels. Hoechst 33258 staining confirmed that ZEA led to apoptosis in sheep follicular granulosa cells. This result is consistent with that of previous studies that showed that ZEA induces apoptosis in chicken granulosa and porcine trophoblast cells [17,31]. There are reports that, in general, pigs and sheep are more susceptible to ZEA infection than poultry [20]. Because susceptibility to ZEA varies between species, perhaps the pathways in which the effect is triggered are different.

To elucidate the molecular mechanism underlying the toxic effects of ZEA on sheep follicular granulosa cells, bioinformatics analysis was performed on 1395 DEGs identified with transcriptomic analysis. GO entry analysis revealed that mitosis and nucleotide binding were the main effects. KEGG enrichment analysis showed that the MAPK and PI3K-Akt pathways were in the core position. Based on the results of previous experiments, we focused on biological processes related to oxidative damage and apoptosis. Based on the transcriptomic data, special attention was paid to the involvement of HMOX1 in 11 biological processes. Protein interactions were analyzed using the STRING database, and the results showed that HMOX1 interacts with apoptosis-related proteins, such as caspase family proteins, as well as oxidation-related proteins such as Gpx3, Gpx7, Gpx8, GCLC, GCLM, KEAP1, and NQO1. The results of previous studies demonstrated that ZEA causes a decrease in the expression of HMOX1 in piglet testis and mouse testis-supporting cells [44,45]; however, little research has been conducted on the effect of ZEA on the expression of HMOX1 in female follicular granulosa cells. Our results show that ZEA significantly increased the mRNA and protein expression of HMOX1 in sheep follicular granulosa cells, which contradicts the results obtained for supporting cells. The expression of HMOX1 potentially increased to resist the oxidative damage in granulosa cells caused by ZEA. HMOX1, a key cytoprotective gene and enzyme, is potentially involved in complex regulation and may be upregulated by multiple factors [46]. Because ZEA triggers different toxic effects in males and females [3], we have speculated that the mechanisms of ZEA toxicity in different reproductive organs may differ; however, further studies are needed to validate this fact.

NAC exerts its antioxidant effects by scavenging excess ROS [40]. Our results elucidate that after NAC scavenges the ROS induced by ZEA, HMOX1 expression was significantly downregulated at both the mRNA and protein levels; similar changes occurred in the expression of Caspase3, a key apoptosis protein. Several studies have validated the association of HMOX1 expression with apoptosis, proliferation, and inflammation [47]. Therefore, the results of transcriptome data analysis validate the role of HMOX1, a key differentially expressed gene, in the regulation of ZEA-induced oxidative stress and apoptosis in sheep follicular granulosa cells. This finding helps to complement studies on the mechanisms of reproductive toxicity of ZEA in different species and may also provide new targets for the screening of toxicity-mitigating drugs.

## 4. Materials and Methods

### 4.1. Isolation and Culture of Sheep Granulosa Cells

All experimental procedures were approved by the Animal Care and Use Committee of Gansu Agricultural University (approval no: GSAU-AEW-2017–0003). Ovaries were collected from female adult small-tailed Han sheep (body weight = 25–30 kg) in a local slaughterhouse of Lanzhou City, Gansu Province. Ovaries were transferred to the laboratory at 33–37 °C in phosphate-buffered saline (PBS) containing 100 IU/mL penicillin and 100 mg/mL streptomycin. After washing the ovaries with sterile PBS, granulosa cells were collected from the follicles with a 5 mL syringe, centrifuged to collect cell precipitates, and inoculated into cell culture flasks. Subsequently, they were cultured in a mixed medium consisting of 89% DMEM/F12 (Gibco, Grand Island, NY, USA) and 10% fetal bovine serum (FBS; BI, Kibbutz Beit Haemek, Israel) and incubated at 37 °C and 5% CO_2_ for 24 h.

### 4.2. Immunofluorescence

Granulosa cells were fixed with 4% paraformaldehyde for 30 min, treated with TritonX-100 for 20 min, and then incubated with BSA for 30 min. Next, cells were incubated overnight with FSHR antibody (1:300; Proteintech) at 4 °C. Then, cells were incubated in darkness with fluorescent secondary antibody at 37 °C for 2 h. Subsequently, the cells were visualized and photographed using a fluorescence microscope (APExBIO, Houston, TX, USA).

### 4.3. Cell Viability Assay

The cell viability was measured using the Cell Counting Kit 8 (CCK8, Invitrogen, Irvine, CA, USA). Briefly, granulosa cells were treated with different concentrations of ZEA (Sigma-Aldrich, St. Louis, MO, USA) for 24 h. Subsequently, 10 µL of CCK8 was added and incubated for 4 h before measuring optical density (OD) at 450 nm.

### 4.4. ROS, MDA, and GSH

Follicular granulosa cells were treated with DMSO and different concentrations of ZEA (20, 40, and 80 μM) for 24 h. The DMSO group served as a control. ROS levels were measured using an ROS detection kit (ROS, Beyotime, Shanghai, China). The relative fluorescence intensity was scanned and quantified using Image-Pro Plus 6.0 (Media Cybernetics Co., Rockville, MD, USA). The GSH and MDA contents were detected using GSH and MDA assay kits (Jiancheng Bioengineering Institute, Nanjing, China), respectively.

### 4.5. Hoechst 33258 Staining

Apoptosis was detected using Hoechst 33258 staining (Beyotime). The cells were fixed in paraformaldehyde, washed, incubated with Hoechst 33258 staining solution for 4 min, and photographed using a fluorescence microscope (Olympus, XB51, Tokyo, Japan).

### 4.6. RNA Extraction, Reverse Transcription, and Transcriptome Analysis

Total RNA of cells was isolated using the TRIzol reagent (Thermo Fisher Scientific, Waltham, MA, USA) following the manufacturer’s instructions. RNA fragments were then reverse-transcribed to create cDNA using SuperScript™ II Reverse Transcriptase (Thermo Fisher Scientific). Finally, paired-end sequencing was performed using an Illumina Novaseq 6000 (LC-Bio Technology Co., Ltd., Hangzhou, China). Each group comprised three biological replicates.

### 4.7. qRT-PCR

The relative mRNA expressions were detected via qRT-PCR analysis using 2× SYBR Green qPCR Master Mix (Selleck, Houston, TX, USA) in a LightCycler 96 Real-Time PCR System (Roche, Basel, Switzerland). The primer sequences are detailed in Table 1. The results were calculated using the 2^−ΔΔCT^ method.

### 4.8. Western Blot

Total protein was extracted from the cells using RIPA cell lysis reagent (Solarbio, Beijing, China). Proteins were separated through electrophoresis using 12% sodium dodecyl sulfate-polyacrylamide gel (SDS-PAGE), electrotransferred to polyvinylidene difluoride (PVDF) membranes, and blocked using 5% skimmed milk. The PVDF membranes were incubated overnight with the primary antibodies against Bax, Bcl2, Caspase3 (Cell Signaling Technology, Danvers, MA, USA), HMOX1, and GAPDH (Proteintech) at 4 °C. Subsequently, they were incubated with the secondary antibodies for 2 h at 37 °C. Protein bands were detected using enhanced chemiluminescence solution. The bands were scanned for gray values using Image-Pro Plus 6.0 (Media Cybernetics Co., Rockville, MD, USA).

### 4.9. Statistical Analysis

Data were analyzed for significance using SPSS (version 22.0; SPSS Inc., Chicago, IL, USA). Student’s *t*-test was used for two-group comparisons, and one-way analysis of variance (ANOVA) was used for multiple comparisons. *p* < 0.05 was considered to be statistically significant.

## 5. Conclusions

In summary, ZEA treatment in primary sheep follicular granulosa cells reduced the cell viability via induction of cellular oxidative stress and activation through Bcl2 family proteins, leading to apoptosis. Transcriptomic sequencing analysis revealed that HMOX1 is a key differentially expressedgene in regulating ZEA-induced oxidative stress and apoptosis in sheep follicular granulosa cells. We speculate that the regulatory mechanism of HMOX1 potentially involves reduced oxidative stress leading to alleviated apoptosis. These experimental data help to complement studies on the mechanisms of ZEA reproductive toxicity in different species.

## Figures and Tables

**Table 1:** Primers for qRT-PCR.

Primers	Primer Sequences	Product Length (bp)
*Bax*	CGCCCTTTTCTACTTTGCCA	92
	CCAATGTCCAGCCCATGATG	
*Bcl2*	CAGTGGGAACCTTTGCGATT	130
	CTCTGCACGCTGGTTGAAAG	
*Caspase3*	ACGTTGTGGCTGAACGTAAA	117
	AGTCCACTGATTTGCTTCCGT	
*CCNA2*	GGACAAAGCTGGCCTGAATC	122
	ATTGACTGTTGTGCGTGCTG	
*CSPG4*	CTGGTCCGGCACAAGAAGAT	109
	AGAACACAATGTCCGCTGGT	
*TGM2*	GCTGTCCGATGCTATGGAGG	127
	CTCCAAGCTGAGGCGGTAAT	
*MYH9*	CCTTCGGGAATGCCAAGACT	98
	GGCTCCCACAATGTAGCCAT	
*CDK1*	AAGTGTGGCCAGAAGTGGAA	122
	TTTCGAGAGCAGATCCAAGCC	
*HMOX1*	CAAGGCACAAGACTCGGCTC	135
	GCATAAAGCCCCACAGCAAC	
*ATG9B*	CTGGCTTCCCTTTCCCGAAT	125
	AGGTAGATGGCGTGGAGACT	
*DEPP1*	CCTCCGTGCTGGATGAAACT	115
	AAGCGAGTGGTGATGTCCTG	
*OSGIN2*	TTAGACTCTCCTGGCCGTCT	106
	TGCCACGCAACTTTCCTTTG	
*AVPI1*	GGCTTCCAGTGAGCAGTATGT	94
	CTGATGGAGGTAGCTTGTGGG	
*GCLC*	AGGACGAACCCAAACCATCC	96
	AGACATGGTCCCACCATACG	

## Data Availability

The data that support the findings of this study are available from the corresponding author upon reasonable request.

## References

[1] Han X., Huangfu B., Xu T., Xu W., Asakiya C., Huang K., He X. (2022). Research Progress of Safety of Zearalenone: A Review. Toxins.

[2] Zinedine A., Soriano J.M., Moltó J.C., Mañes J. (2007). Review on the Toxicity, Occurrence, Metabolism, Detoxification, Regulations and Intake of Zearalenone: An Oestrogenic Mycotoxin. Food Chem. Toxicol..

[3] Knutsen H., Alexander J., Barregård L., Bignami M., Brüschweiler B., Ceccatelli S., Cottrill B., Dinovi M., Edler L., EFSA Panel on Contaminants in the Food Chain (CONTAM) (2017). Risks for Animal Health Related to the Presence of Zearalenone and Its Modified Forms in Feed. EFS2.

[4] Yang D., Jiang X., Sun J., Li X., Li X., Jiao R., Peng Z., Li Y., Bai W. (2018). Toxic Effects of Zearalenone on Gametogenesis and Embryonic Development: A Molecular Point of Review. Food Chem. Toxicol..

[5] Al-Jaal B.A., Jaganjac M., Barcaru A., Horvatovich P., Latiff A. (2019). Aflatoxin, Fumonisin, Ochratoxin, Zearalenone and Deoxynivalenol Biomarkers in Human Biological Fluids: A Systematic Literature Review, 2001–2018. Food Chem. Toxicol..

[6] Minervini F., Dell’Aquila M.E. (2008). Zearalenone and Reproductive Function in Farm Animals. Int. J. Mol. Sci..

[7] Malekinejad H., Schoevers E.J., Daemen I.J.J.M., Zijlstra C., Colenbrander B., Fink-Gremmels J., Roelen B.A.J. (2007). Exposure of Oocytes to the Fusarium Toxins Zearalenone and Deoxynivalenol Causes Aneuploidy and Abnormal Embryo Development in Pigs1. Biol. Reprod..

[8] Koraïchi F., Inoubli L., Lakhdari N., Meunier L., Vega A., Mauduit C., Benahmed M., Prouillac C., Lecoeur S. (2013). Neonatal Exposure to Zearalenone Induces Long Term Modulation of ABC Transporter Expression in Testis. Toxicology.

[9] Long M., Yang S., Dong S., Chen X., Zhang Y., He J. (2017). Characterization of Semen Quality, Testicular Marker Enzyme Activities and Gene Expression Changes in the Blood Testis Barrier of Kunming Mice Following Acute Exposure to Zearalenone. Environ. Sci. Pollut. Res..

[10] Lai F.-N., Liu X.-L., Li N., Zhang R.-Q., Zhao Y., Feng Y.-Z., Nyachoti C.M., Shen W., Li L. (2018). Phosphatidylcholine Could Protect the Defect of Zearalenone Exposure on Follicular Development and Oocyte Maturation. Aging.

[11] Hu P., Sun N., Khan A., Zhang X., Sun P., Sun Y., Guo J., Zheng X., Yin W., Fan K. (2021). Network Pharmacology-Based Study on the Mechanism of Scutellarin against Zearalenone-Induced Ovarian Granulosa Cell Injury. Ecotoxicol. Environ. Saf..

[12] Feng Y.-Q., Wang J.-J., Li M.-H., Tian Y., Zhao A.-H., Li L., De Felici M., Shen W. (2022). Impaired Primordial Follicle Assembly in Offspring Ovaries from Zearalenone-Exposed Mothers Involves Reduced Mitochondrial Activity and Altered Epigenetics in Oocytes. Cell. Mol. Life Sci..

[13] Wang Y., Xing C.-H., Chen S., Sun S.-C. (2022). Zearalenone Exposure Impairs Organelle Function during Porcine Oocyte Meiotic Maturation. Theriogenology.

[14] Eppig J.J. (1991). Intercommunication between Mammalian Oocytes and Companion Somatic Cells. BioEssays.

[15] Yu Y.S., Sui H.S., Han Z.B., Li W., Luo M.J., Tan J.H. (2004). Apoptosis in Granulosa Cells during Follicular Atresia: Relationship with Steroids and Insulin-like Growth Factors. Cell Res..

[16] Guo B., Zhang S., Wang S., Zhang H., Fang J., Kang N., Zhen X., Zhang Y., Zhou J., Yan G. (2023). Decreased HAT1 Expression in Granulosa Cells Disturbs Oocyte Meiosis during Mouse Ovarian Aging. Reprod. Biol. Endocrinol..

[17] Zhu Y., Wang H., Wang J., Han S., Zhang Y., Ma M., Zhu Q., Zhang K., Yin H. (2021). Zearalenone Induces Apoptosis and Cytoprotective Autophagy in Chicken Granulosa Cells by PI3K-AKT-mTOR and MAPK Signaling Pathways. Toxins.

[18] Li X., Chen H., Zhang Z., Duan J., Hua R., Li X., Yang L., Cheng J., Li Q. (2022). Isorhamnetin Protects Zearalenone-Induced Damage via the PI3K/Akt Signaling Pathway in Porcine Ovarian Granulosa Cells. Anim. Nutr..

[19] Zhang G.-L., Feng Y.-L., Song J.-L., Zhou X.-S. (2018). Zearalenone: A Mycotoxin With Different Toxic Effect in Domestic and Laboratory Animals’ Granulosa Cells. Front. Genet..

[20] Liu J., Applegate T. (2020). Zearalenone (ZEN) in Livestock and Poultry: Dose, Toxicokinetics, Toxicity and Estrogenicity. Toxins.

[21] Smith J.F., Di Menna M.E., McGowan L.T. (1990). Reproductive Performance of Coopworth Ewes Following Oral Doses of Zearalenone before and after Mating. Reproduction.

[22] Dicostanzo A., Johnston L., Win Dels H., Murphy M. (1996). A Review of the Effects of Molds and Mycotoxins in Ruminants. Prof. Anim. Sci..

[23] Morris C.A., Amyes N.C., Smith J.F., Sprosen J.M., Towers N.R. (2005). Zearalenone Challenge in Sheep: Variation in Ovulation Rate. Proc. N. Z. Soc. Anim. Prod..

[24] Silva I.P., Brito D.C.C., Silva T.E.S., Silva R.F., Guedes M.I.F., Silva J.Y.G., Rodrigues A.P.R., Santos R.R., Figueiredo J.R. (2021). In Vitro Exposure of Sheep Ovarian Tissue to the Xenoestrogens Zearalenone and Enterolactone: Effects on Preantral Follicles. Theriogenology.

[25] Xu Y., Zhang K.-H., Sun M.-H., Lan M., Wan X., Zhang Y., Sun S.-C. (2019). Protective Effects of Melatonin Against Zearalenone Toxicity on Porcine Embryos In Vitro. Front. Pharmacol..

[26] She J., Feng N., Zheng W., Zheng H., Cai P., Zou H., Yuan Y., Gu J., Liu Z., Bian J. (2021). Zearalenone Exposure Disrupts Blood–Testis Barrier Integrity through Excessive Ca2+-Mediated Autophagy. Toxins.

[27] Xu J., Sun L., He M., Zhang S., Gao J., Wu C., Zhang D., Dai J. (2022). Resveratrol Protects against Zearalenone-Induced Mitochondrial Defects during Porcine Oocyte Maturation via PINK1/Parkin-Mediated Mitophagy. Toxins.

[28] Zhang S., Zhou S., Gong Y.Y., Zhao Y., Wu Y. (2020). Human Dietary and Internal Exposure to Zearalenone Based on a 24-Hour Duplicate Diet and Following Morning Urine Study. Environ. Int..

[29] Malekinejad F., Fink-Gremmels J., Malekinejad H. (2023). Zearalenone and Its Metabolite Exposure Directs Oestrogen Metabolism towards Potentially Carcinogenic Metabolites in Human Breast Cancer MCF-7 Cells. Mycotoxin Res..

[30] Yi Y., Wan S., Wang S., Khan A., Guo J., Zheng X., Li H., Sun N. (2021). Scutellarin Protects Mouse Ovarian Granulosa Cells from Injury Induced by the Toxin Zearalenone. Food Funct..

[31] Bai J., Li J., Liu N., Jia H., Si X., Zhou Y., Zhai Z., Yang Y., Ren F., Wu Z. (2023). Zearalenone Induces Apoptosis and Autophagy by Regulating Endoplasmic Reticulum Stress Signalling in Porcine Trophectoderm Cells. Anim. Nutr..

[32] Fink-Gremmels J. (2008). The Role of Mycotoxins in the Health and Performance of Dairy Cows. Vet. J..

[33] Tsang B.K. (2003). Regulation of Cell Death and Cell Survival Gene Expression during Ovarian Follicular Development and Atresia. Front. Biosci..

[34] Oyewole A.O., Birch-Machin M.A. (2015). Mitochondria-targeted Antioxidants. FASEB J..

[35] Adibnia E., Razi M., Malekinejad H. (2016). Zearalenone and 17 β-Estradiol Induced Damages in Male Rats Reproduction Potential; Evidence for ERα and ERβ Receptors Expression and Steroidogenesis. Toxicon.

[36] Venkataramana M., Chandra Nayaka S., Anand T., Rajesh R., Aiyaz M., Divakara S.T., Murali H.S., Prakash H.S., Lakshmana Rao P.V. (2014). Zearalenone Induced Toxicity in SHSY-5Y Cells: The Role of Oxidative Stress Evidenced by N-Acetyl Cysteine. Food Chem. Toxicol..

[37] Ben Salem I., Boussabbeh M., Prola A., Guilbert A., Bacha H., Lemaire C., Abid-Essefi S. (2016). Crocin Protects Human Embryonic Kidney Cells (HEK293) from α- and β-Zearalenol-Induced ER Stress and Apoptosis. Environ. Sci. Pollut. Res..

[38] Alonso-Garrido M., Frangiamone M., Font G., Cimbalo A., Manyes L. (2021). In Vitro Blood Brain Barrier Exposure to Mycotoxins and Carotenoids Pumpkin Extract Alters Mitochondrial Gene Expression and Oxidative Stress. Food Chem. Toxicol..

[39] Feng Y.-Q., Zhao A.-H., Wang J.-J., Tian Y., Yan Z.-H., Dri M., Shen W., De Felici M., Li L. (2022). Oxidative Stress as a Plausible Mechanism for Zearalenone to Induce Genome Toxicity. Gene.

[40] Wang B., Wang Y., Zhang J., Hu C., Jiang J., Li Y., Peng Z. (2023). ROS-Induced Lipid Peroxidation Modulates Cell Death Outcome: Mechanisms behind Apoptosis, Autophagy, and Ferroptosis. Arch. Toxicol..

[41] Liu X., Xi H., Han S., Zhang H., Hu J. (2023). Zearalenone Induces Oxidative Stress and Autophagy in Goat Sertoli Cells. Ecotoxicol. Environ. Saf..

[42] Elmore S. (2007). Apoptosis: A Review of Programmed Cell Death. Toxicol. Pathol..

[43] Elkin E.R., Harris S.M., Loch-Caruso R. (2018). Trichloroethylene Metabolite S-(1,2-Dichlorovinyl)-l-Cysteine Induces Lipid Peroxidation-Associated Apoptosis via the Intrinsic and Extrinsic Apoptosis Pathways in a First-Trimester Placental Cell Line. Toxicol. Appl. Pharmacol..

[44] Long M., Yang S.-H., Shi W., Li P., Guo Y., Guo J., He J.-B., Zhang Y. (2017). Protective Effect of Proanthocyanidin on Mice Sertoli Cell Apoptosis Induced by Zearalenone via the Nrf2/ARE Signalling Pathway. Environ. Sci. Pollut. Res..

[45] Cao L., Zhao J., Ma L., Chen J., Xu J., Rahman S.U., Feng S., Li Y., Wu J., Wang X. (2021). Lycopene Attenuates Zearalenone-Induced Oxidative Damage of Piglet Sertoli Cells through the Nuclear Factor Erythroid-2 Related Factor 2 Signaling Pathway. Ecotoxicol. Environ. Saf..

[46] Hou W.-H., Rossi L., Shan Y., Zheng J.-Y., Lambrecht R.W., Bonkovsky H.L. (2009). Iron Increases HMOX1 and Decreases Hepatitis C Viral Expression in HCV-Expressing Cells. WJG.

[47] Ayer A., Zarjou A., Agarwal A., Stocker R. (2016). Heme Oxygenases in Cardiovascular Health and Disease. Physiol. Rev..

